# Predictors of sustained virological response to a 48-week course of pegylated interferon alfa-2a and ribavirin in patients infected with hepatitis C virus genotype 4

**DOI:** 10.4103/0256-4947.51816

**Published:** 2009

**Authors:** Hamad Al Ashgar, Ahmed Helmy, Mohamed Q. Khan, Khalid Al Kahtani, Mohammed Al Quaiz, Mohammed Rezeig, Ingvar Kagevi, Abdullah Alshehri, Abdullah Al Kalbani, Khalid Al Swat, Salim Dahab, Naser Elkum, Mohammed Al Fadda

**Affiliations:** a From the Section of Gastroenterology, Department of Medicine, Research Centre, King Faisal Specialist Hospital and Research Centre, Riyadh, Saudi Arabia; b From the Department of Biostatistics, Epidemiology and Scientific Computing, Research Centre, King Faisal Specialist Hospital and Research Centre, Riyadh, Saudi Arabia; c From the Department of Tropical Medicine and Gastroenterology, Assiut University Hospital and Faculty of Medicine, Assiut, Egypt

## Abstract

**BACKGROUND AND OBJECTIVES:**

Knowledge of the predictors of sustained viral response (SVR) to pegylated interferon (PEG-INF) alfa-2a and ribavirin (RBV) therapy in patients with hepatitis C genotype-4 (HCV-4) is crucial for selecting patients who would benefit most from therapy. We assessed the predictors of SVR to this combination therapy in Saudi patients with chronic HCV-4 infection.

**PATIENTS AND METHODS:**

This retrospective study included 148 patients with HCV-4 infection who underwent clinical, biochemical and virological assessments before treatment and at 12, 24, 48 and 72 weeks post-treatment.

**RESULTS:**

Of the 148 patients, 90 (60.8%) were males. Mean (SD) for age was 48.5 (12.7) years and BMI was 27.9 (7.5) kg/m^2^. Seventy-nine of 148 (60.1%) patients were treatment naïve and 110 (74.3%) underwent pre-treatment liver biopsy. Eighteen (12.2%) patients did not complete therapy because of side effects or they were lost to follow up. Early virological response was achieved in 84 of 91 (92.3%) patients. In the 130 (87.8%) patients who completed therapy, 34 (26.2%) were non-responders and 96 (63.8%) achieved end-of-treatment virological response (ETVR). SVR and virological relapse (24 weeks after ETVR) occurred in 66/130 (50.7%) and 30/130 (31.2%) patients, respectively. Compared to relapsers, sustained responders were significantly younger (*P*=.005), non-diabetic (*P*=.005), had higher serum albumin (*P*=.028), lower alpha-fetoprotein level (*P*=.026), lower aspartate aminotransferase (AST) (P=.04) levels, and were treatment-naivve (*P*=.008). In a multivariate regression analysis, the independent predictors of SVR were younger age (*P*=.016), lower serum AST (*P*=.012), and being treatment naivve (*P*=.021).

**CONCLUSION:**

Approximately half of HCV-4 patients who complete the course of combination therapy achieve an SVR, especially if they are young, treatment naivve and have lower AST levels.

Chronic hepatitis C virus (HCV) infects approximately 170 million people worldwide, is a major cause of chronic hepatitis, liver cirrhosis and hepatocellular carcinoma and represents the most frequent cause for liver transplantation in the US and Europe.^1^ Moreover, the incidence of chronic liver failure secondary to HCV-related liver cirrhosis is expected to increase over the next 10 years as a result of the ‘silent epidemic’ of HCV infection.^2^

Pegylated interferon (PEG-INF) plus ribavirin (RBV) therapy given for 48 weeks is now established as the standard therapy for patients with chronic HCV infection with genotypes 1 and 4.^3^ This treatment has yielded overall sustained virological response (SVR) rates of 54% to 69% in randomized controlled phase III clinical trials.^4^–^6^ However, response to treatment is not uniform across all populations^7^ and is dependent on various viral and host factors. Most of the studies conducted worldwide have included patients infected with HCV genotypes 1, 2 and 3.^4^–^6^,^8^–^10^ According to these studies, factors independently associated with higher SVR to combination therapy include serum HCV-RNA levels below 2 million copies/mL, body weight <75 kg, age younger than 40 years, an absence of pre-treatment bridging fibrosis or cirrhosis, being treatment naïve, infection with HCV genotype 2 or 3, and favorable initial virological response.^4^–^6^,^8^–^10^

HCV-4 is known to be endemic in Central Africa and in the Middle East.^11^,^12^ However, several recent studies carried out in Europe have indicated changes in genotype distribution and have underlined the increasing prevalence of HCV-4.^13^–^15^ The prevalence of HCV antibody positivity in Saudi Arabia ranges from 1% to 3%,^16^,^17^ with genotype 4 representing 60% to 70% of these infections.^18^–^21^ There are limited reports on the treatment of chronic HCV-4 patients from the Middle East (mainly from Saudi Arabia, Egypt, Kuwait, and Qatar),^22^–^31^ or elsewhere.^32^–^35^ All these studies were heterogeneous and were weakened by small numbers of patients, the use of conventional interferon with or without RBV, different durations of therapy, the inclusion of patients co-infected with human-immunodeficiency virus (HIV), the lack of liver histopathology data and by the absence of data assessing the predictors of SVR. A summary of these studies is shown in Table 1. Also, the only available meta-analysis that has assessed PEG-INF therapy in HCV-4 patients included only 6 studies, 4 of which were in abstract form and the other two included only 65 patients.^36^ Therefore, the primary objectives of this retrospective study were to evaluate the overall efficacy and safety of 48 weeks course of PEG-INF alfa-2a and RBV combination therapy in 148 consecutive Saudi patients with chronic HCV-4 infection and to assess the independent predictors of SVR in these patients.

**Table 1:** Summary of the previous studies that used interferon to treat hepatitis C genotype 4 patients.

Reference [n]	n	Therapeutic regimen used	Duration (weeks)	SVR, n (%)	SVR predictors
Derbala et al 2005^22^	31	INF alfa-2b 3 MU/3× wk + RBV 800-1200 mg/d^a^	48	8 (25.8)	Not tested
	30	PEG-INF alfa-2b 1.5 μg/kg/wk + RBV 800-1200 mg/d	48	10 (33.3)	

el-Zayadi et al 2005^23^	40	PEG-INF alfa-2b 100 μg/kg/wk + RBV 1000-1200 mg/d^a^	48	22 (64.7)	Not tested
	70	PEG-INF alfa-2b 100 μg/kg/wk + RBV 1000-1200 mg/d^a^	24	34 (54.8)	
	70	INF-alfa 2b 3 MU/3× wk^b^ + RBV 1000-1200 mg/d^a^ + AMD 100 mg/d	24	20 (30.3)	

Al-Tarif et al 2005^30^^c^	19	INF-alfa 2b 3 MU/3× wk	48	43 (15.7)	Not tested
	21	INF-alfa 2b 3 MU/3× wk + RBV 1000-1200 mg/d	48	9 (42.8)	

Kamal et al 2005^25^	95	PEG-INF alfa-2b 1.5 μg/kg/wk + RBV 1000-1200 mg/d (24 wk)	48^d^	28 (29)	Tested^e^
	96	PEG-INF alfa-2b 1.5 μg/kg/wk + RBV 1000-1200 mg/d (36 wk)	48^d^	63 (66)	
	96	PEG-INF alfa-2b 1.5 μg/kg/wk + RBV 1000-1200 mg/d (48 wk)	48^d^	66/(69)	

Al-Faleh et al 2004^26^	31	INF alfa-2b 3 MU/3× wk + RBV 800 mg/d	48	10 (32.3)	Not tested
	28	PEG-INF alfa-2b 1.5 μg/kg/wk + RBV 800 mg/d	48	12 (42.9)	

Diago et al 2004^35^	24	PEG-INF alfa-2a 180 μg/wk + RBV 1000-1200 mg/d^f^	48	20 (79)	Not tested
	12	PEG-INF alfa-2a 180 μg/wk + RBV 1000-1200 mg/d	24	8 (67)	
	8	PEG-INF alfa-2a 180 μg/wk + RBV 800 mg/d	48	5 (63)	
	5	PEG-INF alfa-2a 180 μg/wk + RBV 800 mg/d	24	0 (0)	

Hassan 2004^29^	66	PEG-INF alfa-2b 1.5 μg/kg/wk + RBV 800 mg/d	48	45 (68)	Tested^g^

Koshy et al 2002^31^^h^	21	INF alfa-2b MU/3× wk	26	0 (0.0)	Not tested
	26	INF alfa-2b 3 MU/3× wk + RBV 1000 mg/d	21	3 (14.0)	

Sherman 2001^34^	5	INF alfa-2b MU/3× wk OR INF-alfa2b 6 and 3 MU/3× wk (for 12w & 36w)	48	0 (0.0)	Not tested
	11	PEG-INF alfa-2a 180 μg/wk	48	5 (45)	

Zylbergberg 2001^33^	20	INF alfa-2b only	NR	1 (5.0)	Not tested

Bruno 2000^32^	18	INF alfa-2b, 5-6 MU 3× wk + RBV 1000-1200 mg/d	48	2 (11.1)	Not tested

al-Faleh et al 2000^27^^i^	49	INF alfa-2b 3 MU/3× wk + RBV 1000 mg/d	24	6 (12.2)	Not tested
	18	INF alfa-2b 3 MU/3× wk + RBV 1000 mg/d	24	1 (5.6)	

el-Zayadi et al 1999^24^	24	INF alfa-2b MU/3× wk	24	5 (20.8)	Not tested
	25	INF alfa-2b 3 MU/3× wk + RBV 1000 mg/d	24	2 (8.0)	

al-Faleh et al 1998^28^	80	INF alfa-2b 3 MU/3× wk	24	(16)	Not tested

Kamal et al 2007^60^	358	PEG-INF alfa-2b 1.5 μg/kg/wk + RBV 10.6 mg/kg/d	24, 26, and 48	239 (66.8)	Tested^j^

PEG-INF: pegylated interferon, RBV: ribavirin. 3× wk: 3 times per week.

a RBV dose adjusted according to weight.

b Used an induction dose of INF alfa-2b 3 MU/day for weeks.

c Total number included was 62; 40 (64.5 %) were genotype 4.

d PEG-INF alfa-2b was given for 48 weeks.

e Independent predictors were low viral load, and age ≤40 years.

f Patients received 1000 or 1200 mg of ribavirin on the basis of body weight (<75 kg or >75 kg).

g By univariate analysis for baseline high viral load versus low viral load and whether treatment-naive or previously treated, and according to fibrosis score.

h Patients had cirrhosis.

i Study included 97 patients at the start, but SVR was tested in only 67; group 1 (n=49) previously non-responders to INF alone and group 2 (n=18) treatment-naive cases.

j Study had groups, complex design, and predictors of SVR were older age, higher body mass index, and low baseline viral load.

## PATIENTS AND METHODS

This retrospective study included 148 consecutive patients with chronic HCV-4 infection referred to King Faisal Specialist Hospital and Research Centre (KFSHRC), Riyadh, Saudi Arabia between February 2003 and November 2005. Baseline characteristics of subjects are shown in Table 2. The institutional Research Advisory Council and Research Ethics Committee approved this study. Baseline assessment included clinical history, physical examination, body mass index (BMI), routine hematological, biochemical, serological and virological tests including HCV qualitative and quantitative polymerase chain reaction (PCR) and HCV genotype. Pre-treatment liver biopsy for pathological grading and staging was done in 110 (74.3%) patients. The hepatic inflammation (grade) and fibrosis (stage) in the biopsy specimens were evaluated according to the METAVIR scoring system.^37^

**Table 2:** Patient characteristics (n=148).

Variable	All patients (n=148)	Treatment naïve (n=89)	Pre-treated (n=59)
Age (years), mean (SD)	48.5 (12.7)	46.1 (13.4)	52.2 (10.7)^a^
Sex			
Male	90 (60.8)	54 (60.7)	36 (61.0)
Female	58 (39.2)	35 (39.3)	23 (39.0)
BMI (kg/m^2^), mean (SD)	27.9 (7.5)	27.8 (7.9)	28.1 (7.0)
Genotype 4	148 (100)	89 (100)	59 (100)
Diabetes			
Yes	45 (30.4)	17 (19.1)	28 (47.5)^b^
No	103 (69.6)	72 (80.9)	31 (52.5)^b^
Renal impairment			
Yes	9 (6.1)	7 (7.9)	2 (3.4)
No	137 (3.9)	82 (92.1)	57 (96.4)
Hemophilia			
Yes	4 (2.7)	2 (2.2)	2 (3.4)
No	144 (97.3)	87 (97.8)	57 (96.6)
Alcohol intake			
Yes	4 (2.7)	2 (2.2)	2 (3.4)
No	144 (97.3)	87 (97.8)	57 (96.6)
Previous organ transplant			
Yes	15 (10.1)	8 (9.0)	7 (11.9)
No	133 (89.9)	81 (91.0)	52 (88.1)
Positive liver autoantibodies			
Yes	12 (8.1)	7 (7.8)	5 (8.4)
No	136 (91.9)	82 (92.2)	54 (91.6)
HBV or HIV co-infection^c^			
Yes	29 (19.6)	19 (21.3)	10 (16.9)
No	117 (80.4)	70 (78.7)	49 (83.1)
Liver biopsy	110 (74.3)	63 (70.8)	47 (79.7)

Data are expressed as n (%) unless noted otherwise. BMI: Body mass index. HBV; hepatitis B virus. HIV; human immunodifficiency virus.

a *P*=.004 versus treatment naïve group,

b *P*=.000 versus treatment naïve group.

c HIV is positive in 2 patients only.

Patients were then treated with PEG-INF alfa-2a (40 KD; Pegasys, F. Hoffmann-La Roche, Basel, Switzerland, 180 microgram weekly) plus RBV (Copegus, F. Hoffmann-La Roche, Basel, Switzerland, 1000-1200 mg daily) for 48 weeks. Clinical, biochemical and viral parameters were collected both pre-treatment and at weeks 12, 24, 48 and 72 of follow-up.

Serum HCV RNA was extracted using an automated extraction system. HCV detection and quantification were performed using an Abbott Real-Time M2000rt PCR assay, which utilized two sets of primers and probes and targeted a conserved region of the 5′ untranslated region of the genome and an internal control. This assay detects and quantifies HCV genotypes (1-6) with a detection limit that ranges from 30 to 100 000 000 IU/mL, where 1 IU/mL=4 copies/mL. Prior to treatment, HCV genotype was performed in all patients (n=148; 100%) using INNO-LiPA HCV II (Innogenetics NV, Ghent, Belgium).^38^ Real-time PCR has been available in our institution since January 2006, but the lower detection limit and the unit used have changed in the last 1 to 2 years.

Before 2006, viral load testing was performed using the Bayer Quantiplex bDNA System (Bayer Corp, Tarrytown, NY, USA). The lower quantification detection limit was 3200 copies/mL. The highest detection limit was 40 000 000 copies/mL. The average period between the quantitative PCR test and the start of therapy was 2.7 months. The National Institute of Health guidelines state a drop of ≥2 log10 in serum HCV viral load is indicative of response. An early viral response (EVR) was defined as ≥2 log10 drop in serum HCV viral load at 12 weeks after start of treatment. An end-of-treatment virological response (ETVR) was defined as an undetectable serum HCV RNA at 48 weeks. A sustained viral response (SVR) was defined as a persistently undetectable HCV RNA at 72 weeks (6 months after the end of course of treatment). Nonresponse (NR) was defined as a persistent positive HCV (PCR) after 48 weeks of treatment.

Data were collected initially in a specialized data collection form, then introduced into a Microsoft Excel worksheet and finally transferred to the Statistical Package for Social Sciences (SPSS) version 15.0 for Windows (SPSS 15.0, SPSS Inc., Chicago, IL, USA) for analysis. Means of continuous variables were compared using *t* tests or non-parametric tests (Wilcoxon and Mann Whitney), as appropriate. The chi-square or Fisher exact tests were used to compare frequencies and proportions. Multivariate stepwise logistic regression analysis was performed to determine the independent predictors of sustained response. An intention-to-treat analysis was used. Patients who discontinued treatment and those who did not complete their course of treatment either due to adverse effects or loss to follow-up were not included in the analysis for ETVR or SVR.

## RESULTS

**Figure 1:**
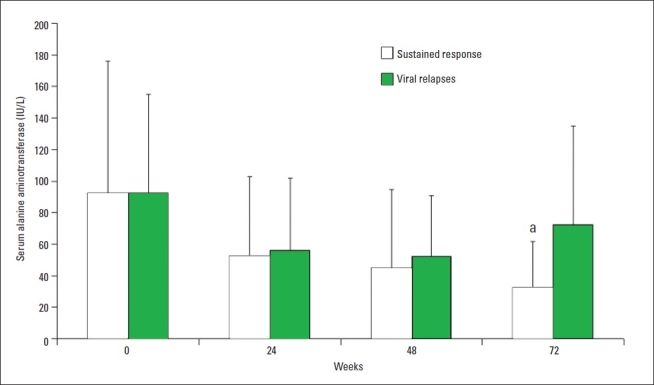
Serum alanine aminotransferase (mean, standard deviation) in patients with sustained virological response (n=66) and in those who relapsed after end-of-treatment response (n=30) at baseline, 24, 48, and 72 weeks ost-treatment. ^a^*P*=.001 vs. viral relapses.

Of 148 patients with HCV-4, 90 (60.8%) were males. Mean and standard deviation for age was 48.5 (12.7) years and BMI was 27.9 (7.5) kg/m^2^. Diabetes mellitus, prior interferon-based therapy, and concomitant HBV or HIV infection were present in 45 (30.4%), 59 (39.9%) and 29 (19.6%) patients, respectively (Table 2). Pre-treatment liver biopsy was done in 110 (74.3%) patients. BMI was similar among diabetics and non-diabetics (27.5 and 28.1 kg/m^2^ respectively; *P*=.66). Steatosis in the liver biopsy was similar in the diabetic group (9 of 33) versus non-diabetics (16 of 78) (P=.33). Sustained responders and viral relapsers had similar serum ALT (alanine aminotransferase) at time points apart from week 72, in which those who relapsed after ETVR showed significantly higher serum ALT (*P*=.001) (Figure 1).

PCR at 12 weeks post-treatment was done in 131 (91.9%) patients from the whole cohort (n=148) and in 91 (94.8%) of those who completed the full course of treatment (n=96). EVR was achieved in 96 patients (64.9%) in the whole cohort (n=148) and in 84 (92.3%) of those who achieved ETVR. EVR was significantly more common in patients who received treatment for the first time compared to those who previously received treatment (61 of 80, 76.3% versus 35 of 56, 62.5%, *P*=.016) (Table 3). However, there was no statistically significant difference between the patients who achieved SVR and those who relapsed after ETVR in the rate of EVR (*P*=.157).

**Table 3:** Clinical and pathological characteristics of sustained responders versus relapsers after end-of-treatment response (ETVR) by univariate analysis.

Variable	Patients with SVR (n=66)	Relapse after ETVR (n=30)	*P* value
Age≥40 years	41 (62.1)	26 (86.7)	.015
Sex M/F	42 (63.6)/24 (36.4)	16 (53.3)/14 (46.7)	.34
BMI≥27 kg/m^2^	34 (51.5)	13 (46.4)	.87
Diabetes mellitus	11 (16.7)	13 (43.3)	.005
Renal impairment	4 (6.7)	2 (6.1)	.91
Previous interferon	17 (25.8)	16 (53.3)	.008
Alcohol intake	1 (1.5)	1 (3.3)	.56
Organ transplant	5 (7.6)	4 (13.3)	.37
HBV or HIV coinfection	9 (13.6)	4 (13.3)	.99
Hemophilia	0 (0.0)	1 (3.3)	.14
Overlap syndrome^a^	7 (10.6)	0 (0.0)	.064
Inflammation grade^b^			
0-2	37 (80.4)	19 (73.1)	.471
3-4	9 (19.6)	7 (26.9)	
Fibrosis stage^b^			
0-2	33 (71.7)	16 (61.5)	.373
3-4	13 (28.3)	10 (38.5)	
EVR^c^	58 (95.1)	26 (86.7)	.157

Data are expressed as n (%). NS; not significant, INF; interferon. EVR; early virological response. SVR; sustained virological response.

a Means detection of ≥1autoantibody in serum.

b Liver biopsy done in 72 patients of the 96 who completed the treatment, 46 in the SVR group and 26 of those who had virological relapse after ETVR.

c PCR at 12 weeks post-treatment was done in 91 patients of the 96 who completed the treatment, 61 in the SVR group and all the 30 who had virological relapse after ETVR.

**Figure 2:**
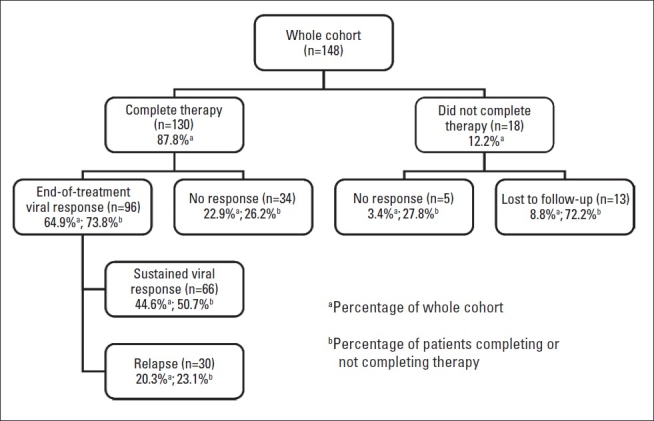
Disposition and virological responses in the whole cohort (n=148).

**Figure 3:**
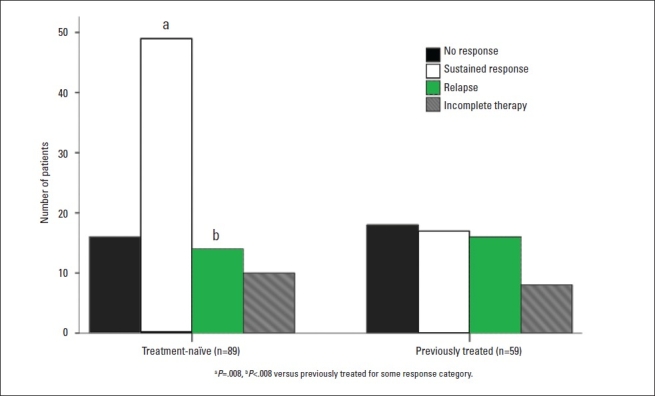
Virological responses in treatment-naïve patients and in those who were previously treated with interferon-based therapy.

Eighteen patients (12.2%) failed to complete the 48-week therapy due to side effects or loss to follow up. The full course of therapy was given to 130 (87.2%) patients; 96 (72.9%) achieved ETVR and 34 (26.2%) were non-responders. SVR was achieved in 66 of 130 (50.7%), while the remaining 30 (31.2%) developed virological relapse after ETVR (Figure 2). Virological responses in treatment-naïve patients and in those who were previously treated with interferon-based therapy are shown in Figure 3. By univariate analysis, treatment-naïve patients had a significantly higher SVR (*P*=.008) and lower relapse rate after ETVR (*P*<.008) compared to those who were previously treated with interferon-based therapy. However, the difference in EVR rate between treatment-naïve patients and those who were previously treated by interferon-based therapies did not reach statistical significance (*P*=.083) (Table 3). Because the study was retrospective and most patients who gave a history of previous INF-based therapy received their initial treatment in other institutions or outside Saudi Arabia, the data on whether the previously treated patients were nonresponders or relapsers was grossly inadequate and difficult to analyze.

By univariate analysis, patients with SVR were significantly younger (*P*=.005), had a lower rate of diabetes mellitus (*P*=.005), higher serum albumin (*P*=.028), lower pre-treatment serum aspartate aminotransferase (AST) levels (*P*=.04), lower serum alpha-fetoprotein (AFP) levels (*P*=.026), and were more treatment-naïve (*P*=.008) than patients who developed virological relapse after ETVR (Table 3, 4). Both groups were similar in pre-treatment inflammation grade, fibrosis stage, viral load, alanine aminotransferase (ALT) levels, interferon dose, RBV dose, organ transplant status, presence of overlap syndrome, co-infection with HBV or HIV, and BMI. By stepwise multivariate logistic regression analysis (using the variables that were significant in the univariate analysis), only being younger (age as a continuous variable), having lower AST levels and being treatment-naïve were independent predictors of SVR (*P*=.016, *P*=.012 and *P*=.021 respectively) (Table 5).

**Table 4:** Baseline laboratory and therapeutic data for sustained response versus relapse after end-of-treatment response by univariate analysis.

Variable	Sustained response (n=66)	Relapse after ETVR (n=30)	*P* value
Age (years)	45.5 (13.5)	53.6 (11.6)	.005
BMI (kg/m^2^)	27.2 (5.2)	27.0 (5.8)	
WBC (×10^9^/L)	6.3 (2.1)	5.7 (2.4)	.87
Hemoglobin (g/L)	142.6 (19.7)	137.1 (18.2)	.23
Platelets (×109/L)	256.6 (115.1)	239.1 (101.4)	.21
Prothrombin time (seconds)	13.6 (4.2)	12.9 (1.2)	.48
Bilirubin (μmol/L)	15.3 (20.5)	14.1 (15.2)	.39
Albumin (g/L)	40.0 (3.7)	38.0 (4.6)	.028
ALT (IU/L)	88.4 (89.0)	90.9 (63.3)	.89
AST (IU/L)	59.9 (44.4)	82.3 (56.4)	.04
GGT (IU/L)	90.4 (66.1)	122.5 (95.9)	.94
ALP (IU/L)	108.2 (91.6)	108.3 (40.0)	.99
Creatinine (μmol/L)	109.0 (146.5)	109.6 (156.4)	.99
Cholesterol (mmol/L)	3.2 (1.4)	3.1 (0.9)	.83
AFP (IU/L)	5.4 (45.4)	15.9 (36.5)	.026
TSH (IU/L)	3.3 (6.9)	3.3 (4.6)	.63
Baseline HCV load (copy/mL)	5.4×10^6^ (7.8×10^6^)	3.7×10^6^ (4.9×10^6^)	.30
HCV load (week 12) (copy/mL)	1.1×10^6^ (1.9×10^6^)	0.6×10^6^ (0.7×10^6^)	.33
Ribavirin dose (mg/day)	923.9 (123.3)	906.9 (155.4)	.28
peg-INF dose (μg/week)	177.2 (13.5)	180.0 (0.0)	.27
Ribavirin dose (mg/kg/day)	12.9 (2.6)	12.7 (2.4)	.77
Peginterferon dose (μg/kg/week)	2.5 (0.6)	2.8 (0.9)	.13

Data are expressed as mean±SD. NS: not significant ALT: alanine aminotransferase, AST: aspartate aminotransferase, ALP: alkaline phosphatase, GGT: γ-glutamyl transferase, INR: international normalization ratio, AFP: alpha-fetoprotein, HCV: hepatitis C virus, PEG-INF: pegylated interferon.

**Table 5:** Independent predictors of sustained virological response (SVR) in stepwise multivariate logistic regression analysis.

Variable	Exp(B)	95% confidence interval		*P* value
Age	1.056	1.01	1.10	.016
Previous INF treatment	0.319	0.12	0.89	.021
Aspartate aminotransferase	1.013	1.00	1.02	.012

A total of 158 side effects were encountered in 66 patients (44.6%) during follow-up (Table 6). Effects that occurred in  ≥5% of patients included fatigue, body aches, weight loss, skin rash, anemia, leucopenia and thrombo-cytopenia. Subcutaneous injections of erythropoietin and granulocyte-colony stimulating factor (G-CSF) were used in 7 patients (19.4% of patients who developed anemia) and 10 patients (25.6% of patients who developed leukopenia), respectively. Dose reduction due to side effects occurred in 62 patients (41.9%). Therapy had to be stopped temporarily 64 times in 43 patients (29.1%) and permanently in only 5 patients (3.4%). A total of 13 (8.8%) patients did not complete treatment due to loss to follow up.

**Table 6:** Frequency of main side effects encountered during therapy.

Side effect	Frequency
Fatigue	15 (10.1)
Body aches (myalgia, arthralgia, headache)	10 (6.8)
Weight loss	12 (8.1)
Itching	4 (2.7)
Skin rash	10 (6.8)
Thyroid dysfunction^a^	6 (4.1)
Anemia	36 (24.3)
Leukopenia	39 (26.4)
Thrombocytopenia	11 (7.4)
Others^b^	10 (6.8)

Data are expressed as n (%).

a Thyroid dysfunction, either hypothyroidism or hyperthyroidism.

b Others include fever (n=3), depression (n=3), cough (n=1), drug intolerance (n=1), allergic reaction (n=1), and nephrosis (n=1).

## DISCUSSION

The present study involved the largest cohort of patients infected with HCV-4 to be reported in the literature after treatment with the combination of PEG-INF alfa-2a and RBV for 48 weeks, and shows that this group of patients can no longer be considered “difficult to treat”. Indeed, with the use of this regimen, SVR was achieved in 44.6% of the whole cohort, in 50.8% of those who completed treatment and in 68.8% of those who achieved ETVR. These results are similar to the responses achieved in previous studies that involved cohorts with predominantly genotype 1 and are less than the responses in patients infected with genotype 2 or 3.^36^,^39^,^40^

Only 18 (12.2%) did not complete their course due to either side effects (n=5) or loss to follow up (n=13) and a total of 34 (26.2%) patients were classified as non-responders. This rate of non-response can be accepted if we put into consideration the tertiary nature of our hospital and the inclusion of many complicated cases such as those who failed previous interferon therapy, cases with organ transplantation, and cases copinfected with HIV and/or HBV. The impression that patients infected with HCV-4 respond poorly to interferon-based therapy and are generally “difficult to treat” came from many earlier studies where conventional interferon-alfa was used alone or in combination with RBV (Table 1).^24^,^28^,^30^–^34^ However, the use of PEG-INF alfa-2 and RBV for 48 weeks lead to a substantial improvement in the rate of SVR as evidenced by other studies who used PEG-INF alfa-2b,^23^,^25^,^29^ and PEG-INF alfa-2a,^35^ as was the case in our study.

Neither the fibrosis stage nor the inflammation grade in the pre-treatment liver biopsy was found to be statistically different between sustained responders and those who developed virological relapse after ETVR. This is contrary to what was previously reported by other studies in patients infected with genotype 1^4^–^6^ and genotype 4.^29^ It should be noted that only 72 of the 96 patients who achieved ETVR in the present study underwent a pre-treatment liver biopsy. Also, only 23 patients in our cohort had fibrosis stage ≥3, and only 3 patients had fibrosis stage 4 (cirrhosis). In addition, liver biopsies had not been performed immediately before the onset of therapy. Moreover, in the study by Hassan et al, SVR was less in patients with an advanced fibrosis score, but this was only in a univariate analysis, and no multivariate analysis was performed.^29^ The study of Kamal et al, however, showed that only age of >40 years and pre-treatment viral load of >2 million copies/mL, can independently predict SVR, and not the pre-treatment liver pathology.^25^ We believe that the effect of pre-treatment fibrosis on the SVR to therapy becomes more obvious if comparisons between cohorts with predominantly stage 3-4 are compared with those with predominantly stage 1-2 are made.

Contrary to other reports,^25^ pre-treatment HCV viral load was not found to be a predictor of SVR in our study. It is well-known that viral load fluctuates and a single reading of HCV quantification may not reflect the actual viral load at the time of treatment, especially if we know that viral load was assessed at varying intervals from the onset of treatment. It has also been reported that the differences in interferon response could be secondary to either a difference in the viral virulence and/or replication rate among different HCV genotypes and not the absolute viral load.^40^

The safety profile of the combination therapy of PEG-INF alfa-2a and RBV used in the present study is comparable to what was previously described in the literature.^25^,^29^,^41^ Indeed, only 18 (12.2%) patients did not complete their course of treatment in our study due to the development of side effects, loss to follow-up and/or transfer to liver transplantation or development of decompensated cirrhosis or hepatocellular carcinoma.

The significantly lower SVR in our patients who previously received interferon therapy (28.8%) compared to those who were treatment-naïve (55.1%) is consistent with the results of many studies in both genotype 1 and genotype 4.^27^,^29^,^41^–^43^ Our results were better than those of Shiffman et al^42^ and Mathew et al,^43^ who reported SVR of 12% to 16% in previously treated patients and 24% to 28% in treatment naïve patients. The mechanism(s) underlying this lower response is not known. However, it may be related to the development of an intrinsic or immunological resistance to the direct anti-viral effect of interferon. Interestingly, interferon-inducible protein 10 kDa (IP-10), which is a chemokine produced by hepatocytes that targets T-lymphocytes, natural killer cells and monocytes was recently identified.^44^,^45^ Elevated serum levels of IP-10 before initiation of therapeutic intervention for HCV infection were reported in patients not achieving SVR.^46^,^47^ A recent study confirmed that pre-treatment IP-10 levels predict SVR in patients infected with HCV genotype 1, even in those with higher BMI and viral load.^48^ Thus, assessment of pre-treatment IP-10 may help in identifying patients for whom current therapy is beneficial. This needs to be tested in patients infected with HCV-4.

Better identification of the pre-treatment host or viral factors that can identify which patients respond better to therapy is currently attracting more attention. For instance, HCV-4 subtyping has been proposed to affect the response to PEG-INF alfa-2a plus RBV combination therapy.^49^–^50^ Other predictors under investigation include increased baseline insulin resistance and AFP levels.^51^,^52^ We have shown that diabetes mellitus and AFP levels were less in sustained responders by univariate analysis. However, neither were found to be independent predictors by multivariate regression analysis. These factors need to be assessed in HCV-4 patients in well-designed prospective studies.

This study demonstrates for the first time that lower baseline serum AST and not ALT is an independent predictor of SVR to PEG-INF alfa-2a and RBV in patients with chronic HCV-4. We believe that these lower AST levels reflect less severe histological parameters in the sustained responders. A study by Zechini et al showed a statistically significant positive correlation of baseline aminotransferase values with the hepatitis activity index and fibrosis score.^53^ In support of our results, a study by Assy et al reported a significant positive correlation between AST values and the extent of hepatic fibrosis.^54^ We compared our patients who had fibrosis stage 0-2 (n=86) and those who scored 3-4 (n=24) for all baseline parameters and found that only younger age and lower AST levels are independent predictors of fibrosis stage 0-2.

Unlike the situation in most randomized controlled trials, the patients included in this study were heterogeneous: 10.1% had previous organ transplantation, 8.1% were positive for liver autoantibodies (classified as overlap syndrome), 19.6% were positive for HBV or HIV serology, 39.9% were non-responders to previous interferon-based therapy and 6.1% had renal impairment. These factors are known to affect the natural history of chronic HCV infection. However, this sample of patients represents what we usually face in real life. If we exclude cases with all the above co-morbidities, our results will only be applicable to patients with isolated HCV infection. As shown in Table 3, sustained responders and relapsers after ETVR were similar regarding transplantation status, BMI, autoantibody status, renal function and HBV or HIV status.

The potential limitations of the current study include the fact that post-treatment biopsy was not done as our main objective was to assess pre-treatment predictors of SVR. There is a solid evidence that SVR is associated with improved outcomes,^55^ stabilization, and/or regression in hepatic fibrosis stage in response to treatment, especially if it associated with viral clearance.^56^–^58^ In addition, assessing the impact of therapy on liver histopathology was beyond the scope of this study. Due to the retrospective nature of the present study, baseline liver biopsy was performed in the majority, but not all cases. Obviously, a better assessment of the predictive role of these two parameters can be done if they were performed in all patients. However, the similarity of the SVR achieved in the present study to what has already been reported by others makes it less likely to substantially affect the results. Another limitation is that patients were followed up for 24 weeks after completion of therapy and thus longer term clinical outcomes could not be determined. Indeed, it has been reported that late relapse may occur after 4 years of completion of interferon therapy.^59^ However, by definition SVR is the persistence of the ETVR for 24 weeks, which was assessed in this study. In addition, early viral kinetics at week 4, which is called rapid virological response (RVR) was not assessed in this study. Indeed, a recently published work from Egypt showed that the duration of combination therapy with PEG-INF alfa-2b and RBV can be shorter treatment for patients who have attained RVR or EVR.^60^ However, the concept of RVR was not entertained at the beginning of our study. This needs to be confirmed in another prospective trial. We have recently published a study involving 335 patients with HCV infection of all genotypes.^61^ It showed that the response of genotype 4 patients to combination therapy is more or less similar to genotype 1. Both showed a worse response compared to those infected with genotypes 2 or 3.

In conclusion, combination therapy with PEG-INF alfa-2a and RBV, if tolerated and completed, is effective in treating chronic HCV-4 patients especially if they are younger than 40 years of age, have no previous interferon therapy and have lower pre-treatment AST levels. Attempts to improve adherence to therapy and the early detection together with treatment of complications are needed to achieve better response to therapy. Further studies addressing other potential predictors of SVR in chronic HCV-4 patients such as the IP10, insulin resistance, HCV-4 subtype heterogeneity are warranted.
